# Primary retroperitoneal angiosarcoma: A case report

**DOI:** 10.1515/biol-2022-0546

**Published:** 2023-02-28

**Authors:** Bin-Qiong Chen, Wei-Wen Luo, Wen-Jie Lin, Wei Zhuang, Shi-Lin Li

**Affiliations:** Department of Ultrasound Medicine, The Second Affiliated Hospital of Fujian Medical University, No. 34 Zhongshan North Road, Licheng District, Quanzhou, 362000, China; Department of Urology Surgery, The Second Affiliated Hospital of Fujian Medical University, Quanzhou, 362000, China

**Keywords:** angiosarcoma, primary, retroperitoneal

## Abstract

Angiosarcoma is a rare subtype of soft tissue sarcoma with identifiable vascular differentiation. It can occur at any age and develop throughout the body, but it is most commonly found in skin, soft, and breast tissues. Primary retroperitoneal angiosarcoma is rarely reported in the relevant literature. This article reports a case of primary retroperitoneal angiosarcoma in a middle-aged man, with the relevant literature reviewed in detail. A 46-year-old male had experienced left waist pain for 2 months. An ultrasonic examination revealed a mass in the left retroperitoneum, and left retroperitoneal lesions were confirmed via computed tomography (CT) and magnetic resonance imaging (MRI). The tumor was removed surgically, and the CT scan revealed local tumor recurrence after 1 month when the first adjuvant therapy was performed. The patient died of a massive hemorrhage from a ruptured tumor. Angiosarcoma has high malignancy and a poor prognosis. Its early diagnosis and treatment significantly impact the long-term survival rate of patients.

## 1 Background

Primary retroperitoneal sarcoma is a malignant tumor originating from mesenchymal tissue in the retroperitoneal space. These tumors are rare, with an incidence rate of only 0.0027% [1]. Among the various types, liposarcoma is the most common, while malignant peripheral nerve sheath tumors and angiosarcomas are rarer. This article reports a case of primary retroperitoneal angiosarcoma in a middle-aged male.

## 2 Case presentation

A 46-year-old male patient was admitted to The Second Affiliated Hospital of Fujian Medical University with the main complaint of left waist pain for 2 months. Previously, he had been in good health, with no underlying diseases and no tobacco or alcohol addiction. A physical examination revealed no bulges in either kidney region, the kidneys were not palpable, and the kidney areas were without percussion pain. Traveling along the bilateral ureteral area, there was no tenderness or palpable mass. Furthermore, there were no obvious abnormalities in the patient’s routine blood and urine tests and biochemical results, while the blood catecholamine, cortisol, and aldosterone readings were normal. A ultrasonic examination revealed no abnormality in the bilateral kidneys and adrenal glands. No obvious signs of metastasis were found in the lung and liver on computed tomography (CT) examination.

**Figure 1:**
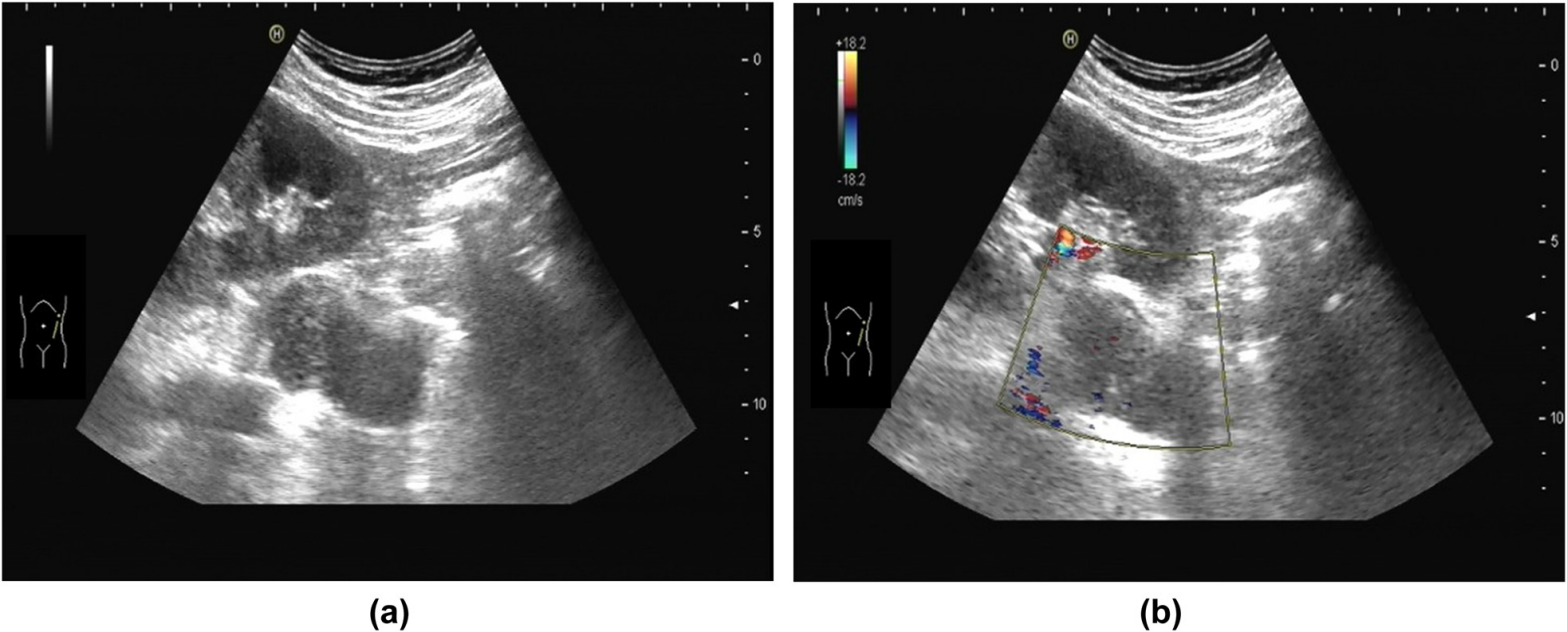
(a) Ultrasonic image: mass in the left retroperitoneum with an irregular shape and uneven internal echo. (b) Ultrasonic image: the CDFI shows no blood flow signal of the mass.

**Figure 2:**
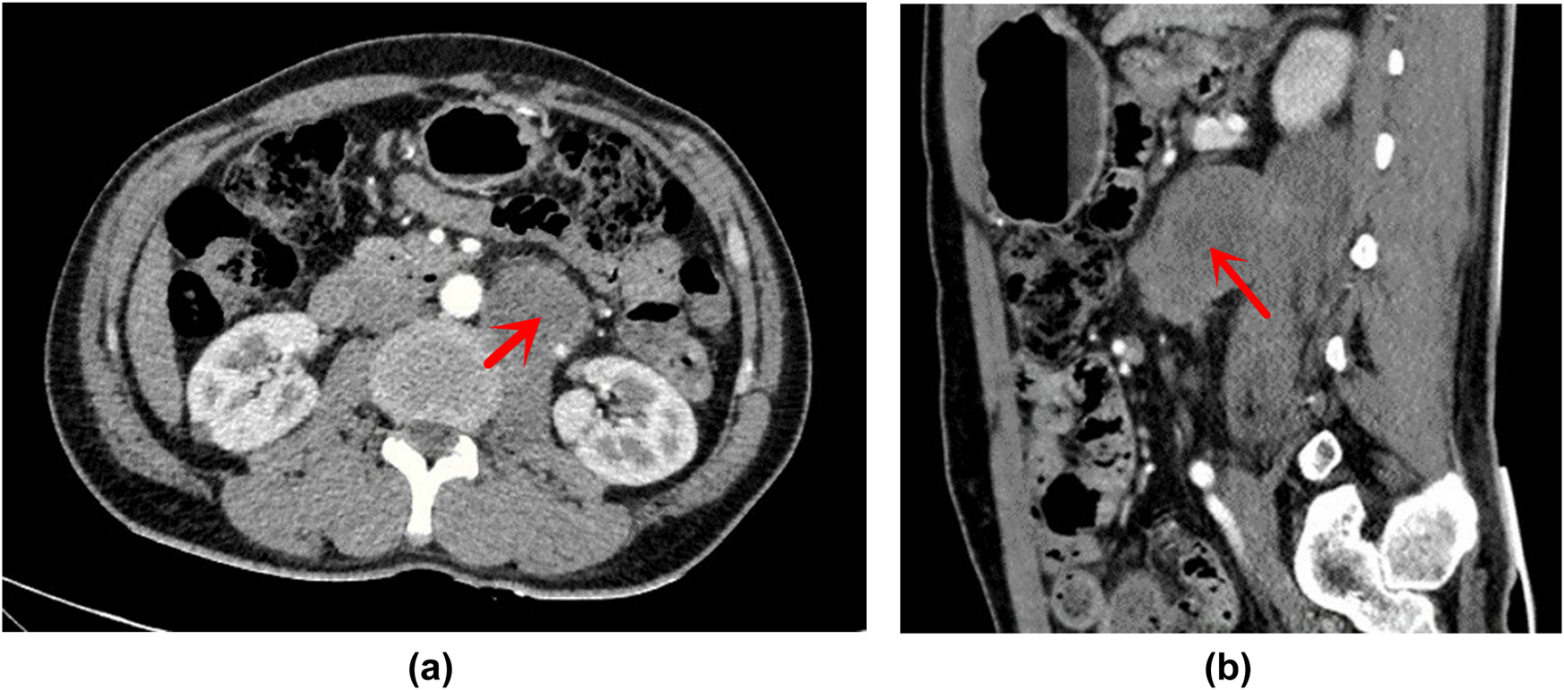
(a) Computed tomography (CT) cross section: low-density area of soft tissue mass in the front edge of the left retroperitoneal psoas major muscle. (b) The CT sagittal plane: low-density area of soft tissue mass in the front edge of the left retroperitoneal psoas major muscle.

**Figure 3:**
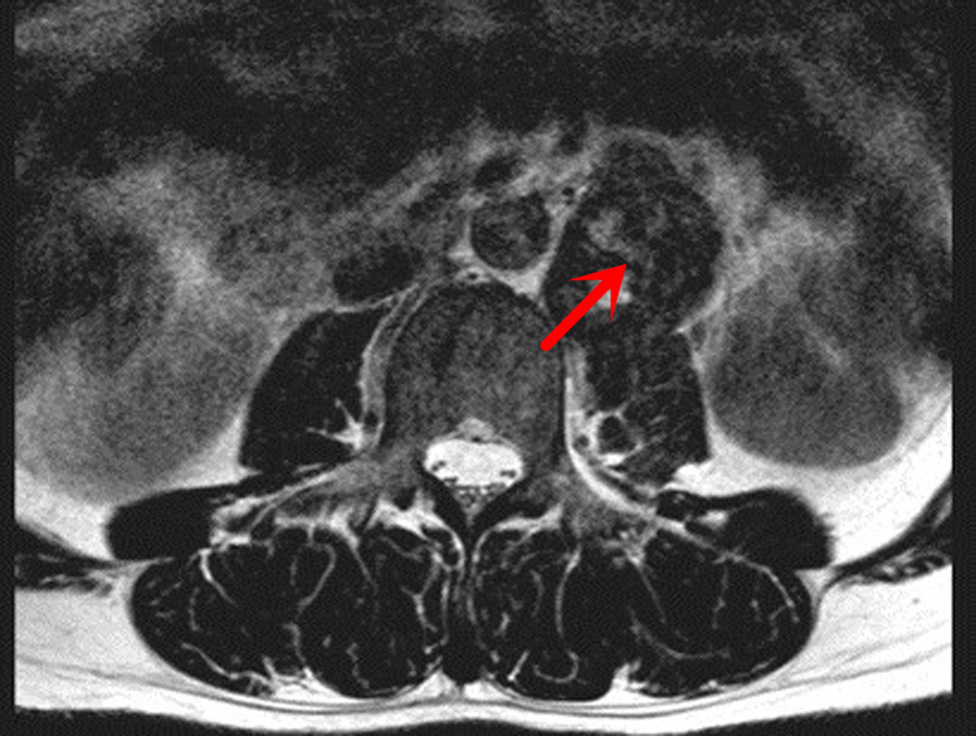
MRI T2WI cross section: quasi-circular abnormal signal area in the left front of the second and third lumbar vertebrae. The T2WI signals were mixed and had uneven enhancement.

The summary of the ultrasonic examination is as follows. The left posterior peritoneum, which was at the height of the left renal artery and abdominal aorta, had an irregularly shaped hypoechoic area (4.3 cm × 4.8 cm × 3.6 cm) with a non-uniform internal echo (Figure 1a). The color doppler flow imaging (CDFI) revealed the internal blood flow to be non-abundant (Figure 1b). The ultrasound diagnosis considered the solid lesions of the left retroperitoneum–retroperitoneal space. The abdominal CT imaging revealed that the anterior edge of the left psoas major muscle was occupying some space, and the possibility of schwannoma (Figure 2a and b) was considered. Magnetic resonance imaging (MRI) of the lumbar spine indicated that the tumor was at the height of the neurogenic lesions of the left front of the L2–L3 vertebral body (Figure 3).

**Figure 4:**
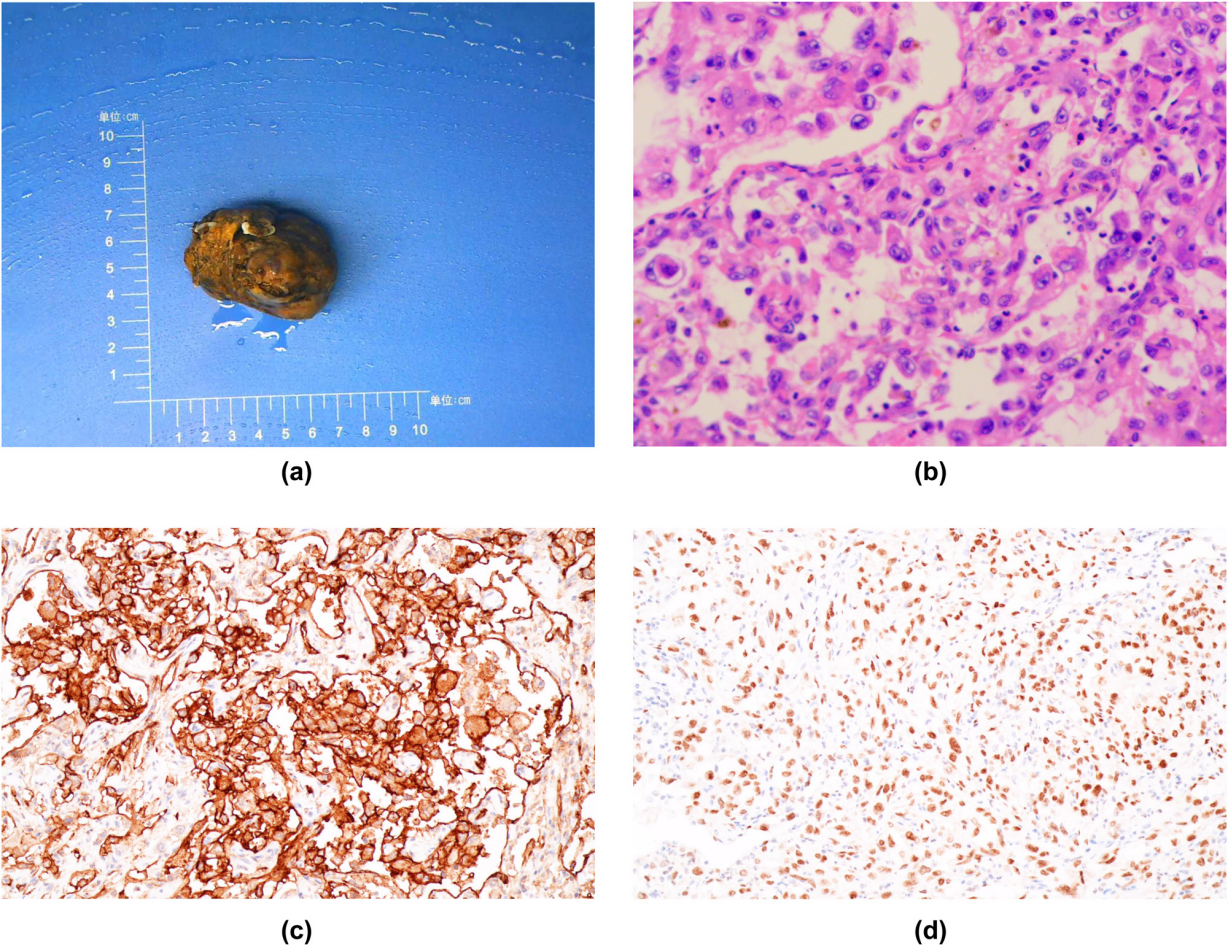
(a) Image of gross specimen: most of the left retroperitoneal tumors are grayish yellow and necrotic. (b) Histopathological image: the left retroperitoneal mass is a malignant tumor with necrosis. Tumor cells are epithelioid, with obvious cell atypia, and mitotic images are easy to visualize (hematoxylin and eosin stain × 200). (c) Immunohistochemical tumor CD31 (+). (d) Immunohistochemical tumor cell ERG (+).


The tumor was removed surgically. During the operation, there was a tan mass of approximately 5 cm × 6 cm below the level of the renal pedicle of the left retroperitoneum. The peripheral blood vessels were distended, but the blood pressure did not fluctuate when the tumor was touched. The boundary of the mass was unclear, and it adhered closely to the psoas muscle. The pathological examination indicated that the left retroperitoneal mass was approximately 6.2 cm × 4.5 cm × 4.2 cm in size. The surgical section of the mass was smooth, with a grayish-yellow necrotic area and a local grayish-white (Figure 4a). The histopathological examination revealed that the left retroperitoneal mass was a malignant tumor with local tissue necrosis within the mass. The tumor cells were epithelioid cells with obvious cytoatypia and clear mitosis (Figure 4b). The immunohistochemistry revealed positive reactivity for pan-cytokeratin (partial), vimentin, cluster of differentiation 31 (CD31), Ets related-gene (ERG), CD34 (minimal), and cytokeratin 7 (on individual cells). However, the tumor was non-reactive for various markers (i.e., epithelial membrane antigen, S100, transcription factor SOX-10, human melanoma black**-**45, melan-A, podoplanin, CD117, discovered on gastrointestinal stromal tumor 1, thyroid transcription factor-1, chromogranin A, and smooth muscle actin). The antigen Ki-67 index was approximately 50% (Figure 4c and d).

**Informed consent:** Informed consent has been obtained from all individuals included in this study.
**Ethical approval:** The research related to human use has been complied with all the relevant national regulations, institutional policies, and in accordance with the tenets of the Helsinki Declaration, and has been approved by the authors’ institutional review board or equivalent committee.

## 3 Discussion

Angiosarcoma is an aggressive malignant endothelial cell tumor that originates from lymph or blood vessels, and its incidence accounts for around 1% of all soft tissue sarcomas [2]. The pathological manifestations of angiosarcoma vary greatly according to the grading of different tumors. For example, low-grade angiosarcoma often presents as a small solid lesion with low cytology and abundant perforating blood vessels. Conversely, the presentation of high-grade angiosarcoma often involves dense cells, high mitotic rates, and atypical cells [3,4]. Angiosarcoma can occur in any part of the body in people of all ages; however, this type of tumor is more commonly found in older individuals (reported median age of 60–71 years). It is most often found in the skin, which accounts for approximately 50% of cases, and only around 10–15% occur in the retroperitoneum [5].

The patient in question has a rare case of primary retroperitoneal angiosarcoma. The etiology of angiosarcoma has not yet been clarified. A history of radiation therapy, chronic lymphedema, and exposure to chemicals, such as vinyl chloride, has been linked with the development of the condition [6]. The patient has no history of radiation therapy or chronic lymphedema. Recent studies have indicated that radiation plays an important role in the receptor protein tyrosine kinase pathway, especially in the upregulation of MYC, KIT, and RET and the downregulation of cyclin-dependent kinase inhibitor 2C [7].

The clinical manifestation of angiosarcoma depends mainly on the site of occurrence. Superficial tumors often appear as fast-growing soft tissue masses. In contrast, deep angiosarcomas can be difficult to detect in the early stage, often being identified only after symptoms of compression and/or pain. If left untreated, an angiosarcoma tumor can grow rapidly (up to 20 cm or larger), and the tumor may rupture and hemorrhage [8]. The CT scan of the patient revealed local tumor recurrence 1 month after the first adjuvant therapy was performed. This suggests that recurrence rates could be high, and close follow-ups with frequent imaging are mandatory [6]. The patient died of a massive hemorrhage from a ruptured tumor. This confirmed that angiosarcoma is an aggressive malignancy that has a poor prognosis [6].

This case supports the view of several studies that a tumor size of more than 5 cm is associated with a worse outcome [8]. The laboratory studies of the patient were generally unremarkable, which is consistent with the findings of previous studies [6]. Imaging examinations lacked specificity in the diagnosis of angiosarcoma. A CT, MRI, or positron emission tomography scan is required to define the anatomy of the tumor and the extent of its spread [9]. The related literature has reported the ultrasonic examination of angiosarcoma to reveal the following [2,10]: (i) a rapidly growing painless mass; (ii) growth (elliptical or lobular) parallel to the long axis of the limb; (iii) an unclear part of the boundary, with no obvious space-occupying effect; (iv) mostly hypoechoic internal echoes (some are potentially detected in strips or masses, and their structure is distorted, caused by multiple fibrin and collagen deposits in angiosarcoma that have separated and freely anastomosed to form vascular channels); (v) probe extrusion can detect mass deformation; and (vi) a CDFI that reveals that most tumors have abundant blood flow signals, and the Adler blood flow classification is grades II–III.

The CT examination of angiosarcoma reveals the following: (i) soft oval tissue masses, some of which may be lobulated; (ii) an unclear boundary with a density much lower than that of muscle; and (iii) possible hemorrhage, necrosis, and calcification occurrence inside the mass.

The MRI examination of angiosarcoma cases reveals the following: (i) T1 weighted imaging (T1WI) produces an uneven low signal, and (ii) T2 weighted imaging (T2WI) produces a high-signal imaging that shows facilitated water movement and a scattered low signal. However, the gold standard for the diagnosis of angiosarcoma is pathological immunohistochemistry, with the expression of platelet endothelial cell adhesion molecule-1 or recombinant cluster of differentiation 34 (CD34) and factor VIII-related antigen (+) characterizing the disease [11,12]. Since an image-guided percutaneous biopsy causes little damage, has a low risk of needle trajectory transfer, and can collect sufficient diagnostic information, it is the most effective diagnostic method for the disease, and it was the first method to provide a histological diagnosis [13]. Due to the particularity of the location, a retroperitoneal puncture is the preferred route. When the retroperitoneal route cannot reach the tumor, the transperitoneal route can be used. Generally, a biopsy does not involve open or laparoscopic surgery. Exposed sarcoma increases the risk of metastasis, may damage important blood vessels and nerves, and changes its anatomical structure, which makes it impossible to provide diagnostic two-dimensional or three-dimensional images [13,14].

Retroperitoneal angiosarcoma needs to be differentiated from the following diseases: (i) pheochromocytoma (most patients with pheochromocytoma have a history of hypertension and rapid changes in blood pressure; most masses are dense and prone to cystic degeneration and necrosis); (ii) retroperitoneal lipoma (the masses are mostly oval or lobulated and soft, with regular edges and complete capsules, the inside of the mass may have a low-to-medium echo, and some may have a high cord-like echo; there is no obvious change in echo behind the mass, and a CDFI reveals no obvious blood flow signal); and (iii) neuroblastoma, which is more common in children; the echo of the mass is much lower than that of angiosarcoma.

Surgical resection is the main method used in the traditional treatment of angiosarcoma. However, the median overall survival of patients with resection of the lesion alone is only 9 months, and the 5-year survival rate is only 20%. For patients supplemented with radiotherapy and chemotherapy, the median overall survival rate can reach 36 months, with a 5-year survival rate of up to 45%. The main causes of death with the disease are recurrence (local recurrence rate: 75%) and distant metastasis (rate: 34%). A multidisciplinary treatment approach is always warranted, but despite multimodality treatment, the recurrence remains high even for localized tumors [15]. Recent data suggest that immune checkpoint inhibition may be active in patients with angiosarcoma [16], while this is yet to be tested in a prospective trial [17].

In summary, angiosarcoma is a rare, fast-growing, and highly aggressive tumor with a very poor prognosis that can occur in any part of the body. The early diagnosis of retroperitoneal lesions is particularly difficult. Clinically, ultrasound, CT, MRI, and other imaging examinations should be integrated. An image-guided needle biopsy is particularly important since it can provide an early diagnosis. To improve the long-term survival rate of patients, a prompt surgical resection of the lesion should be combined with targeted radiotherapy and chemotherapy.

## 4 Conclusion

Angiosarcoma has high malignancy and a poor prognosis. Its early diagnosis and treatment significantly impact the long-term survival rate of patients. This study focused on the imaging manifestations of angiosarcoma; however, there remains a lack of understanding of the disease’s operation mode, radiotherapy, and chemotherapy. Therefore, additional similar cases must be identified and their characteristics summarized to inform further research.
